# Steroidal Triterpenes of Cholesterol Synthesis

**DOI:** 10.3390/molecules18044002

**Published:** 2013-04-04

**Authors:** Jure Ačimovič, Damjana Rozman

**Affiliations:** Centre for Functional Genomics and Bio-Chips, Faculty of Medicine, Institute of Biochemistry, University of Ljubljana, Zaloška 4, Ljubljana SI-1000, Slovenia; E-Mail: jure.acimovic@mf.uni-lj.si

**Keywords:** sterols, steroidal triterpenes, cholesterol synthesis, post-squalene pathway

## Abstract

Cholesterol synthesis is a ubiquitous and housekeeping metabolic pathway that leads to cholesterol, an essential structural component of mammalian cell membranes, required for proper membrane permeability and fluidity. The last part of the pathway involves steroidal triterpenes with cholestane ring structures. It starts by conversion of acyclic squalene into lanosterol, the first sterol intermediate of the pathway, followed by production of 20 structurally very similar steroidal triterpene molecules in over 11 complex enzyme reactions. Due to the structural similarities of sterol intermediates and the broad substrate specificity of the enzymes involved (especially sterol-Δ^24^-reductase; DHCR24) the exact sequence of the reactions between lanosterol and cholesterol remains undefined. This article reviews all hitherto known structures of post-squalene steroidal triterpenes of cholesterol synthesis, their biological roles and the enzymes responsible for their synthesis. Furthermore, it summarises kinetic parameters of enzymes (*V_max_* and *K_m_*) and sterol intermediate concentrations from various tissues. Due to the complexity of the post-squalene cholesterol synthesis pathway, future studies will require a comprehensive meta-analysis of the pathway to elucidate the exact reaction sequence in different tissues, physiological or disease conditions. A major reason for the standstill of detailed late cholesterol synthesis research was the lack of several steroidal triterpene standards. We aid to this efforts by summarizing commercial and laboratory standards, referring also to chemical syntheses of meiosis-activating sterols.

## 1. Introduction

**Scheme 1:**
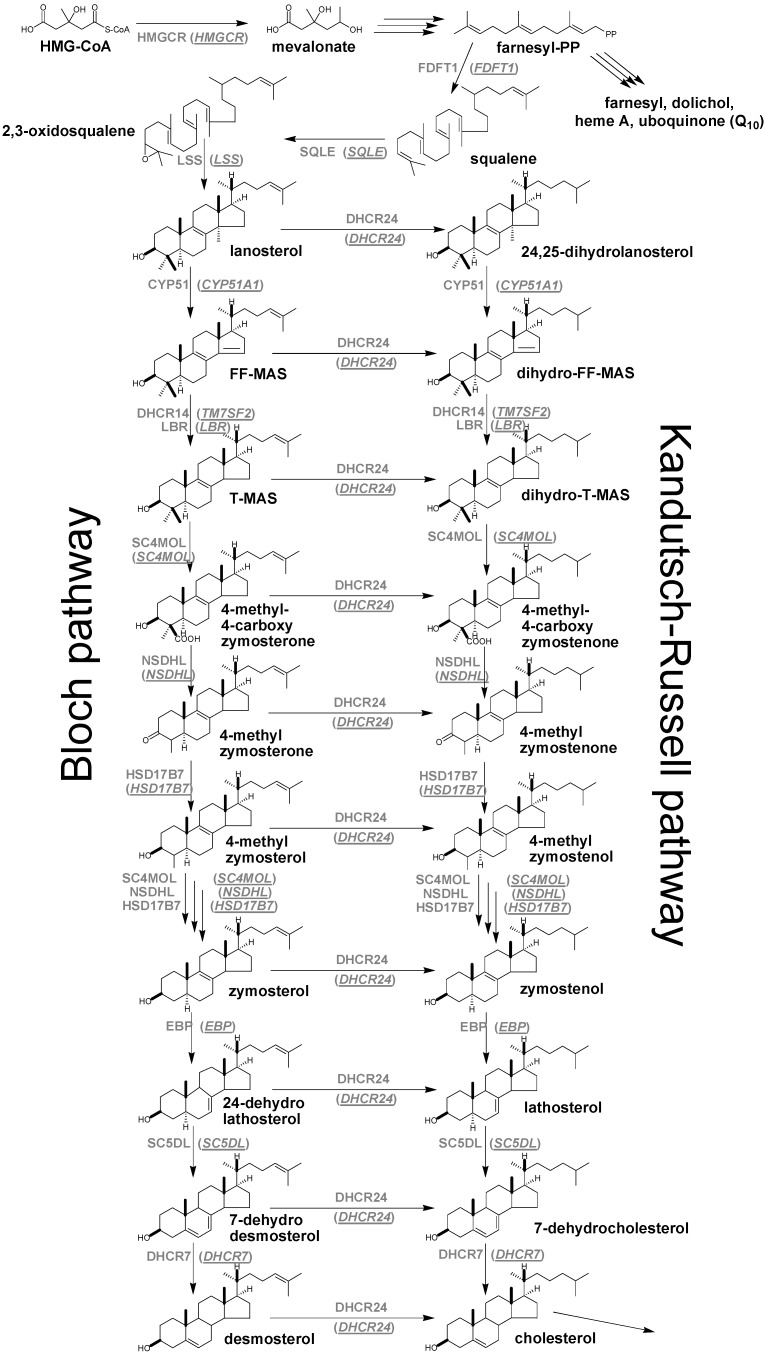
Cholesterol synthesis scheme. For abbreviations see Table 1.

Cholesterol synthesis is a housekeeping pathway believed to take place in virtually all cells in mammals where cholesterol molecules are synthesized from C_2_ acetyl residues in more than 20 reactions. The synthesis starts with the acetylated coenzyme A (acetyl CoA) molecule from which all sterol carbon atoms are derived. Acetoacetyl-CoA is formed from two molecules of acetyl-CoA by acetoacetyl-CoA-thiolase and is further combined with one molecule of acetyl-CoA to form 3-hydroxy-3-methylglutaryl-CoA (HMG-CoA) by HMG-CoA synthase. In the next stage HMG-CoA reductase (HMGCR) which is the rate-limiting enzyme of cholesterol synthesis and thus also the target of cholesterol lowering drugs (statins) converts HMG-CoA to mevalonate (Scheme 1). The latter is further metabolised to farnesyl pyrophosphate (farnesyl-PP) by several enzymes. This point presents the first branching of the pathway. One branch serves for further synthesis of cholesterol while the other ends in farnesyl, dolichol, heme A and ubiquinone (Q_10_). The early part of cholesterol synthesis is completed by the combination of two molecules of farnesyl-PP to squalene by the enzyme squalene synthase 1 (FDFT1) [1]. The post-squalene cholesterol synthesis consists of steroidal triterpenes with cholestane ring structures. It starts with lanosterol, the first cyclic intermediate, which is formed from squalene by squalene epoxidase/monooxigenase (SQLE) and lanosterol synthase (LSS). Then follows a series of demethylation, dehydrogenation and isomerisation reactions of lanosterol leading towards the final product cholesterol (Scheme 1). A detailed metabolic reaction network using the information available from the literature [2,3,4,5,6] and from the pathways databases (Kyoto Encyclopedia of Genes and Genomes—KEGG, BioCyc database collection, LIPID MAPS Consortium) is shown in Scheme 1, where the complexity of the post-squalene phase is presented. Initially, the post-squalene pathway has been divided into the Bloch and Kandutsch-Russell branches [5]. In the Bloch branch, the final reaction is the conversion of desmosterol to cholesterol by sterol-Δ^24^-reductase (DHCR24); thus, all intermediates from lanosterol to desmosterol contain Δ^24^ double bonds. In contrast, in the Kandutsch-Russell branch, DHCR24 acts already on lanosterol; thus all intermediates from 24,25-dihydrolanosterol to 7-dehydrocholesterol contain a saturated side chain. Since DHCR24 can, in principle, metabolise any cholesterol synthesis intermediate from lanosterol on, the two branches cannot be treated separately. Study of DHCR24 substrate specificity *in vitro* [7] showed 24-dehydrolathosterol as the most reactive substrate, suggesting that cholesterol synthesis preferentially starts with the Bloch branch from lanosterol to 24-dehydrolathosterol and is then shifted to Kandutsch-Russell branch *via* lathosterol. In this case 7-dehydrocholesterol presents the last intermediate before cholesterol. In the present work we attempted to summarise all hitherto known structures of post-squalene steroidal triterpenes of cholesterol synthesis, their biological roles and the enzymes responsible for their synthesis. The article also summarises commercial and laboratory standards together with syntheses of meiosis-activating sterols (MAS), and the kinetic parameters of enzymes and sterol concentrations from various tissues.

**Table 1:** Enzymes/gene symbols, names and Enzyme Commission numbers.

Enzyme/Gene symbol	Enzyme/Gene name	Enzyme Commission number
HMGCR	3-hydroxy-3-methylglutaryl-CoA reductase	EC:1.1.1.34
FDFT1	farnesyl-diphosphate farnesyl transferase 1	EC:2.5.1.21
SQLE	squalene epoxidase/monooxygenase	EC:1.14.13.132
LSS	lanosterol synthase	EC:5.4.99.7
CYP51A1	lanosterol-14α-demethylase	EC:1.14.13.70
DHCR14 (*TM7SF2*)	sterol-Δ^14^-reductase	EC:1.3.1.70
LBR	lamin B receptor	3930 ^a^
SC4MOL	sterol-C4-methyl oxidase	EC:1.14.13.72
NSDHL	3β-hydroxy-Δ^5^-steroid-dehydrogenase	EC:1.1.1.170
HSD17B7	3β-keto-reductase	EC:1.1.1.270
EBP	sterol-Δ^8-7^-isomerase	EC:5.3.3.5
SC5DL	sterol-C5-desaturase/lathosterol oxidase	EC:1.14.21.6
DHCR7	sterol-Δ^7^-reductase	EC:1.3.1.21
DHCR24	sterol-Δ^24^-reductase	EC:1.3.1.72

^a^ KEGG Entry number (not listed as an enzyme).

## 2. Nomenclature and Numbering

**Figure 1:**
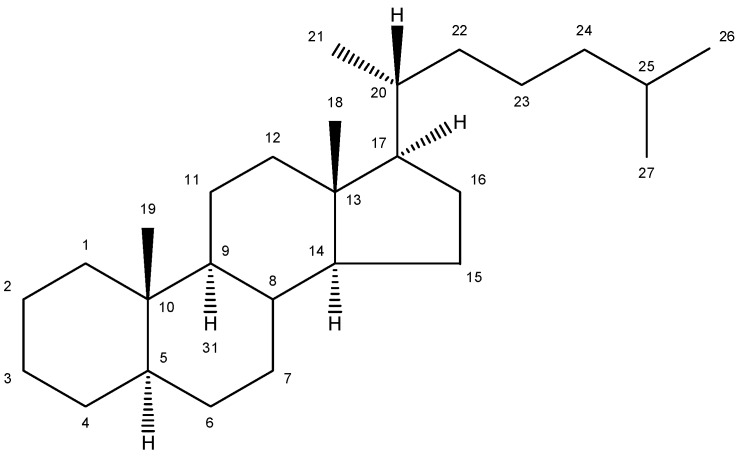
Chemical structure of 5α-cholestane (20*R*) and the IUPAC-IUB recommendations for the atom numbering.

The conventional definition for triterpenes is any isoprenoid having thirty carbon atoms formed from six isoprene (C5-) units. They can be acyclic, as in squalene, or cyclic, as in the tetracycles with a side chain typified by lanosterol [8]. The steroidal triterpenes possess the steroid ring structure and in this article we will refer to the steroidal triterpenes as sterols. The IUPAC-IUB recommendation for numbering of tetracyclic triterpenoid skeleton is shown in Figure 1. 5α-Cholestane is used as a basis for the IUPAC chemical nomenclature in Table 2 [9].

**Table 2:** Sterols of the post-squalene cholesterol synthesis: trivial and chemical names, structures, formulas and molecular weights (Mw).

Trivial name	Chemical name	Structure	Formula	Mw
Lanosterol	4,4,14α-trimethyl-5α-cholesta-8(9),24-dien-3β-ol	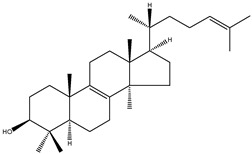	C_30_H_50_O	426.72
24,25-dihydrolanosterol	4,4,14α-trimethyl-5α-cholest-8(9)en-3β-ol	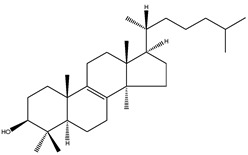	C_30_H_52_O	428.73
FF-MAS	4,4-dimethyl-5α-cholesta-8(9),14,24-trien-3β-ol	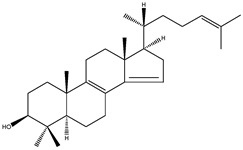	C_29_H_46_O	410.67
Dihydro-FF-MAS	4,4-dimethyl-5α-cholesta-8(9),14-dien-3β-ol	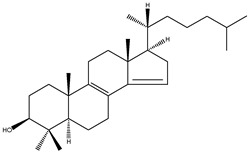	C_29_H_48_O	412.69
T-MAS	4,4-dimethy-5α-cholesta-8(9),24-dien-3β-ol	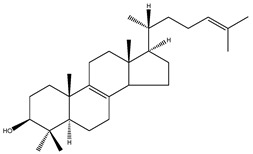	C_29_H_48_O	412.69
Dihydro-T-MAS	4,4-dimethy-5α-cholest-8(9)-en-3β-ol	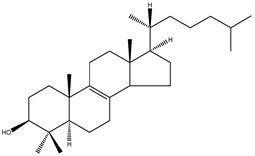	C_29_H_50_O	414.69
Zymosterol	5α-cholesta-8(9),24-dien-3β-ol	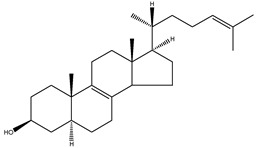	C_27_H_44_O	384.64
Zymostenol	5α-cholest-8(9)-en-3β-ol	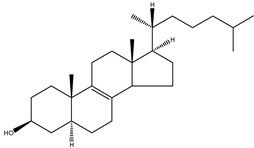	C_27_H_46_O	386.65
24-dehydrolathosterol	5α-cholesta-7,24-dien-3β-ol	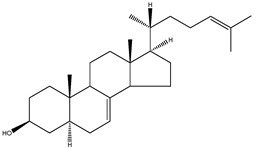	C_27_H_44_O	384.64
Lathosterol	5α-cholest-7-en-3β-ol	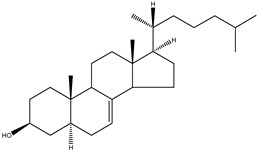	C_27_H_46_O	386.66
7-dehydrodesmosterol	5α-cholesta-5,7,24-trien-3β-ol	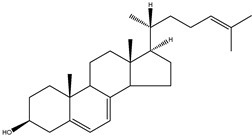	C_27_H_46_O	386.66
7-dehydrocholesterol	5α-cholesta-5,7-dien-3β-ol	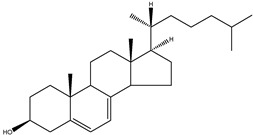	C_27_H_44_O	384.64
Desmosterol	5α-cholesta-5,24-dien-3β-ol	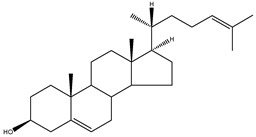	C_27_H_44_O	384.64
Cholesterol	5α-cholest-5-en-3β-ol	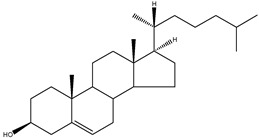	C_27_H_46_O	386.66

## 3. Commercial and Laboratory Standards Together with Some Chemical Syntheses

Availability of sterol standards is crucial for cholesterol synthesis pathway studies. Table 3 summarises the hitherto commercially available sterol standards, including their providers. Lanosterol is readily available from different providers (Table 3) since it is a major constituent (about 15%) of the unsaponifiable portion of wool fat (lanoline) and is thus easily chemically isolated. The first isolation from wool fat was achieved in 1956 by Bloch and co-workers [10]. In 1960 Kandutsch and Russell identified 24,25-dihydrolanosterol, another sterol previously found only in wool fat, in preputial gland tumors inoculated subcutaneously into the axial region of C57BL/g mice. In this manner they obtained relatively large amounts of sterols for characterisation [11]. This study lead to further experiments and ultimately resulted in the discovery of Kandutsh-Russell branch of cholesterol synthesis [5]. 24,25-Dihydrolanosterol standard is also available from some providers (Table 3). 7-Dehydrocholesterol, lathosterol and desmosterol are also commercially available (Table 3), but are more expensive than lanosterol. The first problematic standard is zymosterol. It is listed on the Steraloids web page but upon inquiry you realise that it is actually not available. This situation has existed since 2007 when the chemist responsible for the synthesis of zymosterol retired. Luckily, zymosterol and also zymostenol recently became available from Avanti Polar Lipids (Table 3). This was confirmed by our own inquiry.

**Table 3:** Commercial availability of unlabelled and labelled sterol standards.

Sterol	Providers		
	Unlabelled	Deuterium labelled	Tritium labelled
Lanosterol	APL ^a^, SA ^b^, S ^c^, ARC ^f^	APL	ARC
24,25-dihydrolanosterol	APL, S	APL	ARC
FF-MAS	APL		
T-MAS	APL	APL	
Zymosterol	APL	APL, MI ^d^	
Zymostenol	APL		
Lathosterol	APL, SA, S	APL, MI	
7-dehydrodesmosterol	MI	MI	
7-dehydrocholesterol	APL, SA, S, MI	APL, CIL ^e^	
Desmosterol	APL, SA, S, MI	APL, MI	ARC

^a^ APL, Avanti Polar Lipids (Alabaster, AL, USA; http://avantilipids.com); ^b^ SA, Sigma-Aldrich (St. Louis, MO, USA; http://www.sigmaaldrich.com); ^c^ S, Steraloids (Newport, RI, USA; http://steraloids.com); ^d^ MI, Medical Isotopes (Pelham, NH, USA; http://www.medicalisotopes.com); ^e^ CIL, Cambridge Isotope Laboratories (Andover, MA, USA; http://www.isotope.com); ^f^ ARC, American Radiolabelled Chemicals (St. Louis, MO, USA; http://www.arc-inc.com)

Availability of follicular fluid meiosis-activating sterol (FF-MAS) and testis meiosis-activating sterol (T-MAS) standards presented a problem for the broader research community in the past. FF-MAS and T-MAS were discovered in the laboratory of Prof. Dr. Anne Grete Byskov (The Juliane Marie Center for Children, Women and Reproduction, University Hospital of Copenhagen, Copenhagen, Denmark) who first reported their isolation from follicular fluid and bull semen [12]. In collaboration with Prof. Dr. Ingemar Björkhem (Karolinska Institute, Stockholm, Sweden) we developed a powerful gas chromatography/mass spectrometry method (GC-MS) allowing quantitative analysis of several structurally similar cholesterol precursors (desmosterol, 7-dehydrocholesterol, lathosterol, zymosterol, 24,25-dihydrolanosterol, lanosterol, FF-MAS, T-MAS) in a single chromatographic run. At that time we used the Byskov standards since this was the only resource available to us. We now recommend application and extention of the aforementioned GC-MS method with FF-MAS and T-MAS standards that were recently made commercially available by Avanti Polar Lipids (Table 3). Table 3 also summarises commercially available labelled sterol standards which are necessary for method validation and precise extraction procedures of biological samples. The purity of sterol standards in Table 3 depends on the price. Most of the Avanti Polar Lipids standards are available in more than 99% purity.

In the case that FF-MAS and T-MAS standards would no longer be available from commercial providers they can be extracted from biological samples as described previously [12]. Several chemical syntheses of FF-MAS [13,14,15] have also been reported. A relatively simple, six-step reaction synthesis of FF-MAS from 3β-acetoxy-4,4-dimethyl-5α-cholest-8(14)-en-15-one was published by Ruan *et al*. [13,15] while Murray *et al*. [14] described an alternative synthesis of FF-MAS from lithocholic acid (3α-hydroxy-5β-cholan-24-oic acid) in a ten-step reaction. A six-step chemical synthesis reaction of T-MAS from 3β-acetoxy-4,4-dimethyl-5α-cholest-8(14)-en-15-one was also reported by Ruan *et al*. [13]. Chemical synthesis of dihydro-FF-MAS in a five-step reaction from cholest-4-en-3-one was described by Schroepfer *et al*. [16].

## 4. Enzymes of Post-Squalene Cholesterol Synthesis Pathway and Their Biological Significance

For nearly half a century the remarkable enzymatic cyclization of 2,3-oxidosqualene to form lanosterol (Figure 1) has both fascinated and challenged the molecular sciences. The reaction is catalysed by LSS and a crystal structure of the human membrane protein has been reported [17]. The 2,3-oxidosqualene is stereoselectively cyclised and skeletally rearranged in a single enzyme reaction to form the chair-boat-chair conformation of lanosterol. For a detailed mechanism see Wendt *et al*. [18]. The subsequent enzyme of the post-squalene cholesterol synthesis pathway, lanosterol-14α-demethylase (CYP51), catalyses the oxidative removal of 14α-methyl group of lanosterol and 24,25-dihydrolanosterol to produce FF-MAS or its derivative without the Δ^24^ bond, dihydro-FF-MAS (Figure 1) [19]. The next step is Δ^14^-reduction of FF-MAS or dihydro-FF-MAS into T-MAS or its Δ^24^-reduced derivative dihydro-T-MAS. Two enzymes encoded by two different genes [sterol-Δ^14^-reductase (DHCR14) and lamin B receptor (LBR)] can perform the same enzymatic reaction [20,21]. Formation of zymosterol or its Δ^24^-reduced derivative zymostenol is catalysed by the sterol 4,4-dimethylase complex. First sterol-C4-methyl oxidase (SC4MOL) carboxylates one of the 4-methyl groups of T-MAS or its Δ^24^-reduced derivative and 3β-hydroxy-Δ^5^-steroid-dehydrogenase (NSDHL) coverts the 3β-hydroxy group to a 3-keto one by removal of CO_2_. Finally 3β-keto-reductase (HSD17B7) restores the 3β-hydroxy group to yield 4-methylzymosterol or its Δ^24^-reduced derivative 4-methylzymostenol [6]. The procedure is repeated once more to synthesise zymosterol or zymostenol which are further transformed to 24-dehydrolathosterol and lathosterol by sterol-Δ^8-7^-isomerase (EBP) which catalyses the shift of a Δ^8(9)^ to a Δ^7^ double bond. Sterol-C5-desaturase/lathosterol oxidase (SC5DL) catalyses the formation of the C5-double bond by converting aforementioned sterols to 7-dehydrodesmosterol and 7-dehydro-cholesterol, respectively. Reduction of the Δ^7^ double bond is catalysed by sterol-Δ^7^-reductase (DHCR7) to convert 7-dehydrodesmosterol to desmosterol and 7-dehyrocholesterol to the final product cholesterol [22,23]. Cholesterol is formed from desmosterol by DHCR24 catalysing the reduction of Δ^24^ double bond. As aforementioned (see Introduction) all intermediates of the Bloch pathway which contain an unsaturated Δ^24^ bond, can act as substrates for DHCR24 with 24-dehydrolathosterol having the highest DHCR24 affinity (Table 4) [7]. All enzymes of the post-squalene cholesterol synthesis pathway reside in endoplasmic reticulum being membrane bound [24]. Enzymes and gene abbreviations are listed in Table 1. Enzyme kinetic parameters are listed in Table 4.

**Table 4:** Kinetic parameters of cholesterol synthesis enzymes with specific sterols as substrates.

Enzyme	Substrate sterol	*V_max_* [nmol/min per mg of protein]	*K_m_* [µM]	*k_cat_* [*V_max_*/*K_m_*]	Relative *k_cat_* (fold)	Reference
CYP51A1	Lanosterol	262.3	10.5	24.1		[25]
	24,25-dihydrolanosterol	377.4	20.0	18.87		
DHCR14	4,4-dimethy-5α-cholesta-7,24-dien-3β-ol	1.13	29.4			[26]
EBP	Zymosterol	0.325	25			[27]
SC5DL	Lathosterol	0.017	15.4^a^			[28]
DHCR7	7-dehydrocholesterol	0.85	30			[22]
DHCR24	Lanosterol	0.361	109	0.0033	1.0	[7]
	Zymosterol	3.550	176	0.0201	6.1	
	7-dehydrodesmosterol	2.176	37	0.0586	17.8	
	Desmosterol	2.945	163	0.0180	5.5	

^a^ nmol/mg of membrane protein.

The human malformations and the mouse knock-out models of enzymes from cholesterol synthesis show their high biological importance. In humans, there are six inherited disorders linked to the post-squalene cholesterol synthesis. The Smith-Lemli-Opitz syndrome (SLOS) is the most common in humans. Patients with this syndrome are characterized by significantly reduced plasma, cell and tissue cholesterol levels and elevated concentrations of 7-dehydrocholesterol and its isomer 8-dehydrocholesterol [29], which are a consequence of DHCR7 defects, as DHCR7 is responsible for conversion of 7-dehydrocholesterol to cholesterol. Desmosterolosis, a rare autosomal recessive disease, is characterized by a number of developmental abnormalities and increased desmosterol concentration in serum and tissues as a consequence of *DHCR24* gene mutations [30]. Humans with the mutations in the gene for SC5DL exhibit reduced cholesterol and increased lathosterol levels in plasma, tissues and cell cultures, therefore the disease name lathosterolosis [30,31,32]. HEM dysplasia, the last autosomal recessive disease is caused by the defects of LBR gene, which serves as a sterol Δ^14^-reductase of post-squalene cholesterol synthesis. It is very rare, but when expressed it is lethal in an early stage of embryonic development [33]. CHILD and Conradi-Hunermann-Happle syndrome are inherited in an X-linked dominant fashion. Patients with CHILD syndrome have a defective gene for NSDHL [34] and the Conradi-Hunermann-Happle syndrome is characterized by the defect of Δ^8-7^ sterol isomerase enzyme, due to the mutations in EBP gene [35]. The biological significance of the previously mentioned cholesterol synthesis enzymes is confirmed by different mouse knock-out models as defects are generally lethal in mice. The disorders in humans and corresponding knock-out mouse models are summarised in a recent review [4].

## 5. Biological Role and Toxicity

Sterols are essential components of eukaryote membranes. Their incorporation enhances the packing of acyl chains of phospholipids in the hydrophobic bilayer, increases its mechanical strength, and reduces its permeability [36]. Cholesterol is the sterol found in animal cell membranes. It is also a precursor of bile acids and different steroid hormones such as cortisol, aldosterone, progesterone, estrogen and testosterone. Approximately one quarter of total cholesterol is of dietary origin, while three-quarters of it comes from endogenous synthesis [37]. Using skin fibroblasts obtained from SLOS [3] patients, Tulenko *et al*. [38] demonstrated that the SLOS membrane has increased 7-dehydrochlesterol and reduced cholesterol content and abnormal membrane fluidity. X-ray diffraction analyses of synthetic membranes prepared to mimic SLOS membranes revealed atypical membrane organization and thus have increased membrane fluidity.

Another biological role of post-squalene cholesterol synthesis intermediates is that abnormal concentrations accelerate the degradation of HMGCR. Song and Javitt [39] reported that lanosterol and 24,25-dihydrolanosterol stimulate ubiquitination of HMGCR, although with reduced efficiency compared to 25-hydroxycholesterol (approximately 3- and 10-fold for 24,25-dihydrolanosterol and lanosterol, respectively). Thus, the HMGCR-ubiquitinating activity of the methylated sterols coincides with their ability to diminish steady-state levels of the enzyme. The mechanism is reviewed in more detail by Goldstein *et al*. [40]. Fitzky *et al*. [41] have shown that 7-dehydocholesterol also inhibits cholesterol synthesis by accelerating the degradation of HMGCR protein.

In 1995 two biologically occurring meiosis-activating sterols (MAS) were discovered, FF-MAS and T-MAS [12]. T-MAS concentration in mammalian testes is above 30 µg/g, whereas FF-MAS is present only in trace amounts. FF-MAS is found at high concentrations in the mammalian ovary and T-MAS is also present at half of the FF-MAS concentrations [12,42]. Experiments demonstrated the ability of MAS (FF-MAS, T-MAS, dihydro-FF-MAS and dihydro-T-MAS) to trigger the resumption of meiosis in cultured mouse oocytes *in vitro* [12] but not *in vivo* [43]. In addition, MAS compounds were demonstrated to have positive influence on cytoplasmic maturation, an important process occurring during the final step of oocyte development that enables completion of nuclear maturation, fertilization, and proper early embryo development. In contrast to the initially proposed role of MAS in the resumption of meiosis, it was suggested that they might have another role in meiotic nuclear processes and cytoplasmic maturation; however, the exact mechanism of MAS in female reproduction remains controversial [44].

Spermatogenesis and sperm maturation represents another major biological role of sterols [45]. The development of highly specialised spermatozoa requires extensive remodelling of membrane structure, involving stage specific changes in membrane permeability and fluidity. Desmosterol is the predominant sterol in monkey and rabbit spermatozoa and was found to accumulate preferentially in the sperm tail. The difference in membrane desmosterol composition between sperm heads and tails might underline the different functions of these two structures. Desmosterol has an additional double bond between carbon atoms 24 and 25 compared to cholesterol, and might contribute to membrane fluidity, which is necessary for the motility of flagella [46]. A review of Keber *et al.* [45] gives a more detailed description of sterol roles in spermatogenesis and sperm maturation. Sterol concentrations in various tissues of rats and mice are presented in Table 5. 

**Table 5:** Total sterol concentrations (free + esterified) in various tissues of rats and mice.

Organism	Sterol	Blood plasma [µg/mL]	Liver [µg/g]	Brain [µg/g]	Testis [µg/g]
*Mus musculus *^a^	Lanosterol	0.08–0.86	0.92–7.12	3.2–4.7	4.3–17.8
	24,25-dihydrolanosterol		0.08–1.17	0.10–0.13	
	FF-MAS			1.10–2.15	
	T-MAS		0.61–5.65	4.70–8.50	31–110
	Zymosterol				
	Lathosterol			25.3–30.8	7.6–11.6
	7-dehydrocholesterol	0.22–2.39	0.94–9.31	3.31–7.70	2.6–4.8
	Desmosterol	0.43–2.27	0.78–4.14	29.4–97.8	39–91
	Cholesterol	405–1,997	1,965–6,700	12,500–15,500	1,337–2,829
*Rattus *	Lanosterol		0.75–1.38 ^b^		~4 ^c^
*norvegicus*	24,25-dihydrolanosterol				
	FF-MAS				
	T-MAS		~2 ^c^		~49 ^c^
	Zymosterol				
	Lathosterol		15.5–10.4 ^b^		
	7-dehydrocholesterol		2.63–3.31 ^b^		
	Desmosterol		4.55–5.39 ^b^		~2 ^c^
	Cholesterol	589–856 ^b^	2,041–2,618 ^b^		~1544 ^c^

^a^ Liver values from Acimovic *et al*. [47], brain values from Ali *et al.* [48], blood plasma and testis values from our unpublished data (liver data as mass per wet tissue weight); ^b^ Values from Bjorkhem-Bergman *et al.* [49] (data as mass per wet tissue weight); ^c^ Values from Tacer *et al.* [50] (data as mass per dry tissue weight).

The toxic effects of sterols have been reported by Xu *et al.* [51]. CHO-7 cells which are a sub-line of CHO-K1 cells were grown in medium containing physiological concentrations of desmosterol, lathosterol and lanosterol. Lanosterol supplemented cells not only failed to support growth but also killed the cells whereas lathosterol only failed to support growth and desmosterol showed no toxic effect, which was confirmed by Rodriguez-Acebes *et al.* [52], who showed that desmosterol can replace cholesterol in cell proliferation. Accumulation of lanosterol is therefore considered as toxic. Another toxic effect was reported by accumulation of desmosterol in humans in 1960’s. Triparanol (MER-29), a cholesterol-lowering agent, was marketed in United States from 1959 onwards, but was withdrawn by the U.S. Food and Drug Administration in 1962 because the patients receiving triparanol developed cataracts as a result of notable accumulations of desmosterol [53,54,55].

## 6. Conclusions

Triterpene steroid metabolomics is an important part of lipid metabolomics. In recent years we have learned about many more biological roles of these molecules than had been previously anticipated. For example, the post-squalene portion of cholesterol synthesis was believed to have only one goal: to produce cholesterol. However, we now know of some other biological roles of these intermediates that are not dedicated to cholesterol, such as activation of oocyte meiosis, at least *in vitro*. The cholesterol synthesis pathway seems to hide additional secrets. Recent data show that sterol intermediates can serve as direct substrates for enzymes of steroid hormone synthesis which is stimulating further research in this area. From our own experience, the quantitative sterol metabolomics is not trivial and requires specialized protocols and of course standards that are frequently difficult to obtain. The aim of this manuscript was thus to review the current state of the art and standard resources in triterpene steroid metabolomics with focus on the cholesterol synthesis to facilitate research in this important area.
